# Longer in primary care: a mixed-methods study of the Welsh GP training model

**DOI:** 10.3399/BJGPO.2023.0159

**Published:** 2024-02-21

**Authors:** Dorottya Cserzo, Alison Bullock

**Affiliations:** 1 Children’s Social Care Research and Development Centre, School of Social Sciences, Cardiff University, Cardiff, UK; 2 Cardiff Unit for Research and Evaluation in Medical and Dental Education, School of Social Sciences, Cardiff University, Cardiff, UK

**Keywords:** general practice, training, Wales, mixed-methods research, education, evaluation

## Abstract

**Background:**

A new model of GP training was introduced in Wales, whereby trainees spend 1 year in hospital and 2 years in general practice (the 1+2 model), a change from the previous model of 18 months in each setting.

**Aim:**

To evaluate the 1+2 model of GP training in Wales.

**Design & setting:**

Longitudinal mixed-methods evaluation via repeated surveys and focus groups with GP trainers and trainees across the Welsh training schemes.

**Method:**

Yearly surveys and focus groups were undertaken between June 2020 and December 2022. Quantitative survey data were analysed in SPSS. Qualitative survey data and focus group transcripts were analysed thematically.

**Results:**

Spending more time in general practice was seen as a major benefit. The consensus was that general practice is the best place to learn essential consultation skills. Furthermore, general practice was viewed as a flexible educational setting where knowledge gaps can be addressed. The main concern about the 1+2 model was that trainees would miss experience of key specialties. However, as trainees progressed through the training programme, this concern diminished. All trainees and most trainers thought that the benefits of the 1+2 model outweighed drawbacks.

**Conclusion:**

Spending more time in general practice during GP training appears to improve how prepared trainees felt for practice. Future changes should explore options to enhance hospital experience without reducing time spent in general practice.

## How this fits in

In 2019, Wales introduced a new model of GP training whereby trainees spend 1 year in hospital and 2 years in general practice (the 1+2 model) instead of the previous model of 18 months in each setting. Internationally, there is variation in the time spent in hospital and general practice settings during GP training. In this article, we evaluate the impacts of GP trainees spending more time in general practice settings. We found that increasing general practice experience was well received, although some concerns remained around gaining sufficient hospital experience.

## Introduction

Internationally, GP training comprises a combination of posts in hospital and general practice settings. Typically, GP training in the UK takes 3 years, with trainees undertaking posts in general practice (primary care) and in hospitals (secondary care). Although it is argued that it is preferable if the balance of training time is weighted in favour of general practice since GPs require a distinct skillset, which is not well developed in some hospital practice, this is rarely the case.^1–3^ The traditional model is to spend 18 months in general practice and 18 months in hospitals. In Wales, a new model was introduced whereby GP trainees undertake 1 year in hospital and 2 years in general practice (the 1+2 model). The curriculum goals remained unchanged, with the intention of providing GP trainees with a broad base of hospital experience. From August 2019, 101 GP trainees followed this new model, across five participating schemes. From August 2020, the remaining six schemes in Wales adopted the 1+2 model.

The intention of this study was to evaluate the 1+2 model of GP training in Wales. We began preparations for the evaluation in June 2020 and completed it in December 2022. This article explores trainee and trainer perspectives on the perceived benefits or drawbacks of spending a greater proportion of time in general practice rather than hospital-based placements. We draw on data collected at all stages of the evaluation to summarise views on placements in primary and secondary care before assessing the benefits and drawbacks of the 1+2 model of GP training.

## Method

We adopted a mixed-methods design,^4^ conducting surveys^5^ and focus groups^6^ with trainers and trainees. An overview of participants is given in Table 1. As we were interested to explore whether trainee views changed over the course of the programme, we collected data from them on more occasions than from trainers. Our goal was to follow the pilot cohort through their training, consulting comparator trainees who were at the same stage of training but following the traditional model of training. Surveys were issued to trainees towards the start of the second year of training (autumn 2020), towards the end of year 2 (summer 2021), and at the end of their training (summer 2022).

**Table 1.** Participant overview

	**2020**	**2021**	**2022**	Total
Trainer survey responses	66	118	–	184
Trainer focus group participants	18	–	–	18
Trainee survey responses	43	43	19	105
Trainee focus group participants	37	55	39	131

The trainer surveys achieved a response rate of 14% in 2020 and 36% in 2021. Trainee response rates were difficult to calculate because high numbers of less-than-full-time trainees and numbers taking time-out-of-training meant that we also gathered data from trainees who started earlier than our target cohort but were at the same stage of training at the time of the surveys and focus groups. In 2020 there were 582 trainees in total (across all stages and models of training) and 101 trainees starting on the 1+2 model. Over the course of the evaluation, we gathered survey responses from trainers and trainees in every GP scheme in Wales. Quoted extracts in this article are followed by a code indicating trainer or trainee (TR or TEE), focus group or survey (FG or S), year of data collection, and for focus groups, the number of the group.

The design of the questions for all the data collection instruments was informed by the literature, the intentions of the evaluation, results from previous rounds of data collection where those were available, and feedback from the funders of the study, Health Education and Improvement Wales (HEIW). HEIW is a Special Health Authority within NHS Wales with a leading role in the education, training, and development of the healthcare workforce in Wales.

A list of statements concerning placement quality and experience was included in the 2021 survey, where responders were invited to express their level of agreement or disagreement on a 6-point scale. The statements were derived from open-text responses on the 2020 survey. These statements were repeated in the 2022 survey. The surveys were prepared using Jisc Online Surveys and distributed via email by the general practice team at HEIW. Data from completed questionnaires was exported into Excel and analysed in SPSS version 17.^7^

Focus groups were conducted online using Microsoft Teams,^8,9^ with one focus group in 2022 held in person. Six focus groups with trainers were held in 2020 and 26 focus groups were offered to trainees over the course of the evaluation. These focus groups ranged in size from 3–12 but also included two occasions where only two trainees attended and two with a single trainee. Although offered as focus groups, we note that where only one or two participants joined the meeting, the data gathering on these occasions was more akin to a one-to-one semi-structured interview or paired interview.^10^ On these occasions the range of views discussed was more limited; however, we were able to explore the topics in more detail and including these interviews in the sample allowed us to gather experiences from every participating scheme in Wales. We recorded a total of 13 hours and 28 minutes of discussion, with an average of 25 minutes per focus group. Focus groups and interviews were transcribed verbatim and anonymised. An inductive thematic analysis^11–13^ of the transcripts was conducted using NVivo (version 12 Pro).^14^ The analysis was carried out by DC and refined through discussions between co-authors and HEIW staff.

## Results

We present views on placements in primary and secondary care. This is followed by a report of responder perspectives on benefits and drawbacks of the 1+2 model.

### Primary care placements

Survey results show that trainees had positive experiences in general practice settings. They considered that they were provided with relevant learning opportunities, sufficient support, and had few difficulties arranging study leave (see Table 2). Consideration of both the mean and mode values suggests that the differences between the years were not notable.

**Table 2.** Trainee survey responses to statements about general practice (2021 and 2022)

		Agreement with statement score				
Statement		Disagree (1, 2),% (*n*)	Neutral (3, 4),% (*n*)	Agree (5, 6),% (*n*)	Mean	Mode
I have felt supported in my GP placement(s)	2021	5% (2)	10% (4)	85% (34)	5.3	6
	2022	0	11% (2)	89% (17)	5.6	6
My GP placement(s) provided me with learning opportunities that are relevant to my future career	2021	5% (2)	0	95% (37)	5.6	6
	2022	0	6% (1)	94% (16)	5.6	6
There has been too much focus on service provision in my GP placement(s)	2021	43% (17)	23% (9)	35% (14)	3.5	2
	2022	17% (3)	67% (12)	17% (3)	3.6	4
I had difficulties in arranging study leave in my GP placement(s)	2021	70% (26)	24% (9)	5% (2)	2.2	2
	2022	83% (15)	11% (2)	6% (1)	1.9	2

For presentation, the 6-point scale has been simplified to a 3-point scale and ‘not applicable’ responses excluded. N/A was included as an option for those responders who may yet to have experienced that type of placement.

In 2022 trainees were asked to rate on a 10-point scale how satisfied they were with the amount of time work leaves for family, social, and recreational activities (1 = not at all satisfied; 10 = extremely satisfied). All trainees were on a general practice placement at this stage. The overall satisfaction mean was 7.0, an increase in the mean of 6.6 from the 2021 survey responders. Responders were also asked to rate how much they enjoyed their current placement, on a 10-point scale (1 = not enjoying it at all; 10 = enjoying it greatly). The mean enjoyment score was 7.4; this compares with an overall enjoyment mean of 7.2 from the 2021 survey data (when trainees were on a variety of specialty placements) and for general practice, 7.7.

In terms of skills development, trainers argued that spending more time in general practice enhanced confidence and managing uncertainty, facilitated the development of administration and consultation skills, and chronic disease management. Free-text responses from trainers on the 2021 survey included comments about how greater experience in general practice provided exposure to *‘a wider spectrum of cases and challenges*’, enabling ‘*greater chance to develop many skills needed to be a good generalist*’ (TRS21). Another remarked how trainees could *‘learn more of the admin/management of general practice*’ (TRS21).

In focus groups, trainees highlighted that general practice placements are invaluable in meeting educational needs because of the inherent relevance of all experience and the scope to respond flexibly to trainee needs:

*‘I think that being in general practice* […] *you’re learning from absolutely every patient interaction.’* (TEEFG20 2)

*‘My next GP rotation, I feel like I can say “give me loads of kids to see please”. And most places will try and help you with it.’* (TEEFG22 2)

Trainers held similar views, suggesting that knowledge gaps can be *‘targeted’* in primary care. For example, one remarked on the 2020 survey that ‘*any shortfall in hospital specialties can be targeted in primary care*’ (TRS20).

### Secondary care placements

Views on the value and quality of secondary care placements within GP training were more mixed, with mean response to statements ranging from 3.4 to 4.8 (Table 3). Compared with responses presented in Table 2, higher proportions of trainees thought that there was too much focus on service provision in hospital placements. Similarly, the response to the statement about difficulties arranging study leave in hospital placements (Table 3) were less favourable compared with the responses to the same statement for GP placements (Table 2).

**Table 3.** Trainee survey responses to statements about hospital placements (2021 and 2022)

		Agreement with statement score				
Statement		Disagree (1, 2), % (*n*)	Neutral (3, 4), % (*n*)	Agree (5, 6), % (*n*)	Mean	Mode
I have felt supported in my hospital placements	2021	3% (1)	18% (7)	80% (32)	4.8	5
	2022	5% (1)	32% (6)	63% (12)	4.6	5
My hospital placements provided me with learning opportunities that are relevant to my future career	2021	12% (5)	12% (5)	76% (32)	4.8	5
	2022	0	47% (9)	53% (10)	4.6	4
There has been too much focus on service provision in my hospital placements	2021	10% (4)	45% (19)	45% (19)	3.4	4
	2022	6% (1)	29% (5)	65% (11)	4.5	5
I had difficulties in arranging study leave in my hospital placements	2021	37% (15)	29% (12)	34% (14)	3.6	2
	2022	46% (6)	8% (1)	46% (6)	3.5	4 & 5

For presentation, the 6-point scale has been simplified to a 3-point scale.

Service provision was contrasted with educational needs by trainees and trainers alike. Trainees tended to focus on the impact on their learning:

*‘I feel sometimes in hospital you’re just there to provide a service and you’re just rattling through people without actually learning.’* (TEEFG20 5)

Trainers linked the tensions between education and service provision in hospital posts to the broader context of the NHS. Some thought that the tension was exacerbated by the pressures on the NHS and accepted that this was a necessary part of the training programme:

*‘There’s a little bit too much emphasis on them providing the service than there should be, but that’s because we work in the NHS and it’s under pressure.’* (TRFG20 6)

One trainer thought that hospital placements were ‘*a waste of time*’ for GP trainees and suggested getting ‘*rid of the hospital posts*’ (TRFG20 6). Other trainers suggested a less radical change: focusing on outpatients in hospitals in order to provide a more relevant learning experience.

*‘If trainees spent more time in outpatient clinics then they would be learning about conditions which they would more often encounter in the community.’* (TRS20)

Trainers noted that specialty knowledge can be acquired not only in hospital placements but also by utilising self-directed learning (SDL) time and attending outpatient and other clinics. Some thought that these other avenues of developing specialty knowledge were more relevant to general practice, diminishing the importance of extended hospital placements as a source of specialty knowledge:

*‘Trainees would be better off attending outpatients, endoscopy, theatres. The knowledge and skills gained from this would be more useful for a career in GP* [than hospital placements]*.’* (TRS20)

However, a minority of trainers indicated that spending longer time in general practice might lessen preparedness for practice. For these trainers, hospital placements were fundamental in GP specialty training and reduced exposure to hospital specialties was a notable concern. In their view, lack of experience in hospital specialties led to gaps in clinical knowledge:

*‘Fewer hospital posts in the rotation means more people aren’t exposed to key areas (paediatrics, obstetrics and gynaecology and to a lesser extent psychiatry).’* (TRS21)

*‘It has reduced their exposure to specialties and therefore their clinical experience, meaning there will be more gaps in their clinical knowledge.’* (TRS21)

A concern about ‘*missing out on key specialties*’ (such as obstetrics and gynaecology, paediatrics, emergency medicine, and psychiatry) was also expressed by trainees, especially earlier on in their training.

*‘The drawback* [of the 1+2 model] *is that now people are getting only two hospital rotations. Whereas I had three and what you lose out on is a whole specialty.’* (TEEFG21 09)

### Benefits and drawbacks of spending more time in primary care

When asked about the benefits and challenges of the 1+2 programme, trainees had more to say about the benefits. Participants often expressed that general practice is the best place to learn to be a GP, therefore, more time spent in that setting was valuable:

*‘We’re training to become GPs, so the longer time you have in GP practice, the better.’* (TEEFG22 5)

*‘Learning to be a GP in GP land is more relevant than trying to learn to be a GP in hospital.’* (TEEFG22 3)

*‘The best place to learn to be a GP is in GP!’* (TRS21)

Trainers highlighted that sufficient time in general practice is important because the role is increasingly complex as health needs of the population change:

*‘Trainees need more time in general practice as it is getting more complex* […]*. We are taking new responsibilities, newer roles, eg., managing chronic health conditions, anticoagulation, comorbidities, and 18 months is not enough to have good experience in all modalities of general practice now.’* (TRS20)

In addition to the clinical knowledge, trainees need to learn about existing resources, make links with colleagues, and develop competence in running a business:

*‘I really welcome the change to two years in general practice because there’s so much to learn* […]. *Knowing what community resources are there. Linking in with other professionals.* […] *Understanding how practices work, how they generate income.’* (TRFG20 2)

However, trainers also noted that an increased number of trainees place greater demand on the time and attention of GP trainers, and on the physical space in general practice surgeries:

*‘Ideally would need more rooms/larger premises/more GP trainers. More time in general practice means more trainees in general practice and a lot more burden on surgeries. This is time-consuming and very stressful for trainers.’* (TRS20)

In the surveys, trainees were asked to indicate the extent to which they agreed or disagreed with the statement: *‘I am confident that at the end of my programme I will be prepared to practise as a GP.’* In 2022 the mode was 6 (strongly agree) and all bar one at least slightly agreed (see Table 4). These results represent a small increase in the level of agreement compared with 2021 (when the mode was 5) and those results were very similar to 2020. When responses were cross-tabulated with the model of programme (traditional or 1+2), the pattern was broadly similar. Numbers disagreeing were too small for statistical analysis.

**Table 4.** Trainee survey responses to statements about feeling prepared to practise as a GP and the impact of the 1+2 model

		Agreement with statement score				
Statement		Disagree (1, 2),% (*n*)	Neutral (3, 4),% (*n*)	Agree (5, 6),% (*n*)	Mean	Mode
I am confident that at the end of my programme I will be prepared to practise as a GP	2020	9% (4)	7% (3)	84% (36)	4.8	5
	2021	5% (2)	12% (5)	83% (35)	5.0	5
	2022	5% (1)	16% (3)	79% (15)	5.1	6
A reduction in the number of hospital posts will weaken a trainee’s preparedness for practice	2021	45% (19)	38% (16)	17% (7)	2.9	2
	2022	53% (10)	32% (6)	16% (3)	2.7	1 & 2
Two years in general practice will improve trainees’ preparedness for practice	2021	0	17% (7)	83% (35)	5.2	6
	2022	5% (1)	0	95% (18)	5.3	5 & 6
The benefits of the 1+2 model outweigh the drawbacks	2021	2% (1)	21% (9)	74% (31)	5.1	6
	2022	0	0	100% (19)	5.5	5
GP posts are the best place to develop the knowledge and skills needed for a career in general practice	2021	3% (1)	6% (2)	91% (29)	5.4	6
	2022	0	5% (1)	95% (18)	5.5	6

For presentation, the 6-point scale has been simplified to a 3-point scale.

When asked in 2022 whether a reduction in the number of hospital posts would weaken a trainee’s preparedness for practice, about half disagreed (53% gave a rating of 1 or 2; the percentage disagreeing was lower in 2021, 45%). A complementary statement read *‘two years in general practice will improve trainees’ preparedness for practice*’. The great majority agreed or agreed strongly with the statement in 2022 and the mean was 5.3. The results were similar to the 2021 survey, where the mean was 5.2.

When asked in 2022 whether the benefits of the 1+2 model outweigh the drawbacks, all agreed. This represents an improved response compared to the 2021. All agreed (at least slightly) in 2022 that GP posts are the best place to develop the knowledge and skills needed for a career in general practice. These results are very similar to the previous survey. At the end of the focus group discussions in 2022, we asked trainees to indicate their preferred model of training. Thirty-one trainees preferred the 1+2 model over the traditional model, with only one trainee favouring the traditional model. The focus groups provided further indication that trainees changed their opinion during training increasingly favouring the 1+2 model as they progressed through the placements:

*‘At the beginning of the rotation, I felt a bit apprehensive about the 1+2. Because at that point you feel like you want more hospital postings, like that will prepare you to be a GP. But now in retrospect, I think the 1+2 actually* […] *addresses what you need as a GP trainee.’* (TEEFG22 1)

Trainees highlighted the benefit of gaining experience in an additional general practice placement. Working in more general practice settings allowed them to experience different working cultures, administrative processes, and work with different types of patients. These varied experiences could have a notable impact on how confident they felt about their career choice:

*‘The 1+2 is very good for going to three different GP practices over time.* […] *it puts you in a much more powerful position to know what you want personally. What deprivation group, whether you want to go and work in the wealthy areas or the poor areas.’* (TEEFG22 2)

Trainer views about the impact on the 1+2 model were more ambivalent. The proportion of trainers indicating that benefits of the 1+2 model outweigh the drawbacks dropped from 74% in 2020 to 59% in 2021 (see Table 5).

**Table 5.** Trainer views on the benefits and drawbacks of the 1+2 model

	**2020**		**2021**	
**Do the benefits of the 1+2 model outweigh the drawbacks?**	*n*	%	*n*	%
Yes	49	74.2	70	59.3
No	4	6.1	15	12.7
Undecided	13	19.7	33	28.0
Total	66		118	

The idea of a 4-year training programme was discussed and proved to be a divisive topic among trainers and trainees alike. Trainees who began specialty training immediately after foundation training were receptive to the idea of extended protected training time. Trainees who had experience in other specialties or were less-than-full-time tended to be somewhat resistant to the idea of extending the training programme. Trainers also highlighted that moving to a 4-year training programme would have implications for those on the current 3-year programme.

## Discussion

### Summary

Primary care placements were highly regarded and trainees expressed an overall satisfaction with them and high levels of enjoyment. Trainers had no doubts about the value of these placements. Secondary care placements were described by some as not providing much educational value, and others as being a crucial part of the training programme. Trainee concerns about reduced time in specialty placements lessened over time.

To elaborate, the survey responses indicated that trainees on both types of programmes felt confident that their programme would prepare them for practice, with confidence increasing in the final year of training. All data sources highlighted the benefit of spending more time in general practice. The consensus was that general practice is the best place to learn necessary GP skills such as consultation and chronic disease management. In addition, general practice was seen as a flexible educational setting where knowledge gaps can be addressed. In contrast, there were numerous references to the tensions between service provision and education in hospital settings. Although most participants thought that spending time in hospitals was also important, there was a sense that time spent in general practice was more likely to bring educational benefits.

Nonetheless, the main concern about the 1+2 model was that trainees would miss out on key specialties. At the start of the evaluation, trainees were more concerned about this issue than trainers. However, as trainees progressed through the training programme, the concern about missing out on specialties diminished. One argument made by trainers and trainees in their third year of GP training was that relevant specialty knowledge can be acquired in general practice or developed in self-directed learning time, for example, by attending clinics.

From a trainer perspective, trainees spending more time in general practice increases workload for trainers and puts more strain on resources like physical space. When asked directly whether the benefits of the 1+2 model outweigh the drawbacks, all trainees agreed as did the majority of the trainers. The proportion of trainers who disagreed was higher in 2021 than 2020, but we are unable to ascertain whether this is owing to a decrease in confidence in the programme or responses from different trainers (there were almost twice as many responders in 2021 as in 2020). We did not collect data from trainers in 2022.

Implicit in making an argument for a 4-year programme is the suggestion that a 3-year programme is inadequate. Transition arrangements would need to be put in place to ensure that those on the shorter programme have qualifications and experience equivalent to those on a longer programme.

### Strengths and limitations

This study gathered a wealth of data about trainee views in the first three years of the 1+2 model. In practice, the cohort was difficult to identify owing to the high numbers of trainees who fall out of synch with their starting cohort over the course of training.

Survey responders were a self-selecting sample, which may introduce selection bias.^15^ Focus group trainees consisted of trainees attending training events, which means that views of trainees who were less engaged may not be represented. Trainer response rates were relatively high, but we did not collect data from them in the final year of the study. We gathered views from trainers and trainees in every Welsh GP training scheme.

The final trainee survey received fewer responses than the previous ones, perhaps owing to survey fatigue and the survey being launched very close to the end of the programme. Although the number of survey responders in the final year was small, we have reason to have confidence in the results because trends in our data fit with other studies.^16^ Furthermore, the findings from the focus group analysis complement and support the survey findings.

### Comparison with existing literature

UK studies have demonstrated that GP training in its current form leaves trainees feeling unprepared for some aspects of the role.^17–19^ The work of GPs is increasing in complexity and recruitment of GPs is not keeping pace with demand.^20^ The link between training experience and career choice has been well-established and thus initiatives that extend the training experience in primary care may both enhance preparedness for practice and benefit GP recruitment.^21^

Our participants’ ratings of enjoyment and leisure are similar to a study reported by Surman *et al*.^16^ They found that ratings of enjoyment and leisure were higher for doctors in general practice compared with doctors in other specialties. These findings could be used to encourage GP recruitment.

A supportive learning environment in GP placements is a critical influence on career choice.^22^ Arguably this is especially important at the beginning of the first GP placement, as research has found that the learning curve is initially steep^23^ and learning to tolerate ambiguity has been identified as a central challenge.^24^ Our participants also highlighted the benefits of a supportive training environment and confirmed the increasing complexity of the role.

### Implications for research and practice

Spending more time in general practice during training was welcomed by the majority of our participants. Nonetheless, there were concerns around ensuring that trainees have sufficient clinical expertise in hospital specialties. We recommend exploring options to enhance or tailor experience of secondary care to trainee needs, without reducing time spent in general practice. We note that the growth in recruitment of GP specialty trainees will intensify the workload of trainers in coming years unless mitigating action is taken.

## Data

The dataset relied on in this article is available from the corresponding author on reasonable request.
